# Structural Changes of Sodium Warfarin in Tablets Affecting the Dissolution Profiles and Potential Safety of Generic Substitution

**DOI:** 10.3390/pharmaceutics13091364

**Published:** 2021-08-30

**Authors:** Jan Muselík, Martina Urbanova, Eva Bartoníčková, Jakub Palovčík, David Vetchý, Jiří Czernek, Larisa Janisova, Nadiia Velychkivska, Aleš Franc, Jiří Brus

**Affiliations:** 1 Department of Pharmaceutical Technology, Faculty of Pharmacy, Masaryk University, Palackého třída 1946/1, 612 00 Brno, Czech Republic; muselikj@pharm.muni.cz (J.M.); vetchyd@pharm.muni.cz (D.V.); 2 Department of NMR Spectroscopy, Institute of Macromolecular Chemistry, Czech Academy of Sciences, Heyrovsky sq. 2, 162 06 Prague 6, Czech Republic; urbanova@imc.cas.cz (M.U.); czernek@imc.cas.cz (J.C.); janisova@imc.cas.cz (L.J.); velychkivska@imc.cas.cz (N.V.); 3 Materials Research Centre, Faculty of Chemistry, Brno University of Technology, Purkyňova 464/118, 612 00 Brno, Czech Republic; bartonickova@fch.vut.cz (E.B.); xcpalovcik@fch.vut.cz (J.P.)

**Keywords:** warfarin, solid-state NMR, polymorphism, stability, particle size, bioavailability, generic substitution

## Abstract

At present, the risk of generic substitutions in warfarin tablets is still being discussed. The aim of this study was to assess whether API interactions with commonly used excipients may affect the safety of generic replacement of warfarin sodium tablets. These interactions were observed during an accelerated stability study, and the effect of the warfarin solid phase (crystalline/amorphous form) as well as the API particle size distribution was studied. Commercial tablets and prepared tablets containing crystalline warfarin or amorphous warfarin were used. In addition, binary mixtures of warfarin with various excipients were prepared. The structural changes before and after the stability study were monitored by dissolution test in different media, solid-state NMR spectroscopy and Raman microscopy. During the stability study, the conversion of the sodium in warfarin to its acid form was demonstrated by some excipients (e.g., calcium phosphate). This change in the solid phase of warfarin leads to significant changes in dissolution, especially with the different particle sizes of the APIs in the tablet. Thus, the choice of suitable excipients and particle sizes are critical factors influencing the safety of generic warfarin sodium tablets.

## 1. Introduction

Hypercoagulation is among the most common causes of morbidity and mortality in industrialized countries, and is now appearing worldwide in connection with medication and vaccination for COVID-19 [1]. Maximum attention is given to its prevention and treatment. At the beginning of the new millennium, there were high expectations for the introduction of novel oral anticoagulants (NOAC), which were intended to replace warfarin; until then, warfarin had been the most widely used anticoagulant. NOACs were intended to reduce the frequency of International Normalized Ratio (INR) monitoring, reduce the risk of interactions between drugs and food, reduce the incidence of major bleeding, and accelerate the onset of action [2]. However, these expectations have not been confirmed [3,4,5]. Despite the introduction of stricter rules for NOAC treatments that further reduce the risks [6], warfarin is still the drug of choice for many physicians; therefore, its use has also been discussed in the context of COVID-19 [7]. This trend was intensified by the finding that warfarin provides better protection against heart attacks than some promising NOACs [8,9]. Recently, several other comparative studies have been performed that have not clearly shown that NOACs are safer than warfarin in all indications [10,11,12]. Even from an economic point of view, it cannot be stated that NOACs are always more economical [13]. For this reason, warfarin is still a massively prescribed anticoagulant, especially in the US and for elderly patients [14].

However, warfarin has risks and shows not only high interindividual but also intraindividual variability [15]. The substance has a narrow therapeutic index (NTI), where small differences in dose can lead to severe changes in blood clotting, especially to states of increased bleeding. Therefore, there is an effort to evaluate substitutions in generic warfarin tablets more thoroughly [16,17], and there have been restrictions in some states [18]. One method of increasing its safety is to produce high-quality dosage forms. This is because the dosage form can have adverse effects on the safety of the treatment, especially due to the lack of uniformity of content [19]. Many manufacturers, therefore, use stricter parameters for the content uniformity of warfarin-containing tablets than pharmacopoeia require [20]. The decreased stability of APIs in relation to the safety of warfarin treatment has also been discussed in the literature [21,22].

Warfarin is available as warfarin sodium (WS) in drug formulations because of its physicochemical properties, especially solubility, that are considerably better than those of its acidic form (WA). Therapeutically used warfarin sodium (dose 5 mg) is a highly soluble and permeable drug [23] that belongs to class 1 in the biopharmaceutics classification system (BCS), while the warfarin acid form is a low-soluble drug. Additionally, warfarin sodium exists in amorphous (WSA) and crystalline forms (WSC). WSC is a clathrate molecule in which isopropanol (IPA) is entrapped in the crystal lattice of warfarin. USP defines a molecular ratio of 2:1 between warfarin and IPA for WSC. The transition of metastable WSC to WSA during storage has already been described in the literature [24]. However, the danger of generic confusion of these two forms, as shown by clinical meta-analyses [25], and the risk of WSC converting to WSA in the dosage form during its shelf life [26] have not yet been confirmed. These findings correspond to the fact that both forms of warfarin sodium (crystalline and amorphous) rapidly dissolve in the stomach fluid due to the acidic environment. Subsequently, nearly instant transformation of molecularly dissolved warfarin sodium into its acidic form occurs in the dissolved state and thus has no impact on drug absorption. In addition, the crystalline and amorphous forms of sodium warfarin have virtually identical dissolution under absorption modeling conditions. Therefore, the content of the amorphous or crystalline form of sodium warfarin in tablets does not present a serious risk in terms of treatment safety [27].

From a safety point of view, the transition of solid sodium warfarin (WSA or WSC) to the poorly soluble, non-ionized acidic form WA after expiration can be strongly problematic. As mentioned above, if the transition to WA occurs in gastrointestinal tract (GIT) conditions, all doses of warfarin are consumed, thus having no impact on treatment efficacy. However, if the warfarin acidic form (WA) arises in the solid state (in a tablet), then the resulting properties are strongly affected by the size of the WA particles. In particular, due to the extremely low solubility of WA in the solid state, the size of particles has a significant effect on the dissolution rate and bioavailability.

The purpose of this work is to comprehensively characterize the solid-state forms of warfarin in commercial and model drug formulations. Specifically, we focus on interactions of solid warfarin sodium with excipients and transitions of warfarin sodium to its acidic forms under the conditions of the accelerated stability study. We are convinced that multinuclear ss-NMR spectroscopy and Raman microscopy combined with measurements of dissolution profiles and particle size distribution provides a more detailed picture of warfarin behavior in solid-state and tablet formulations.

## 2. Materials and Methods

### 2.1. Materials

#### 2.1.1. Preparation of Tablets

For the evaluation of warfarin solid-state stability, commercial tablets “A” from a Czech market were used, and tablets “B” and “C” were prepared. Tablets “A” contained 5.40 mg of warfarin sodium isopropanol clathrate per tablet, which was equivalent to 5.00 mg of warfarin sodium. The excipients for “A” were lactose monohydrate, cellulose microcrystalline, corn starch, colloidal silicon dioxide and magnesium stearate. Tablets “A” were packed in PVC/Al clear blister (10 tablets in blister and five blisters in a paper box). For the preparation of the “B” tablets, warfarin sodium isopropanol clathrate (WSC; Pliva, Zagreb, Croatia) was used (5.40 mg in one tablet). For the preparation of “C” tablets, the amorphous form of warfarin sodium (WSA) was used (5.00 mg in one tablet). The common excipients of “B” and “C” were Di-Cafos^®^ 92-14 70% (calcium hydrogen phosphate, Budenheim KG, Germany), Avicel^®^ PH 101 25% (microcrystalline cellulose, FMC BioPolymer, USA), Ac-Di-Sol^®^ 2% (sodium croscarmellose, FMC BioPolymer, Philadelphia, PA, USA) and magnesium stearate 1% (Peter Greven GmbH & Co. KG, Bad Münstereifel, Germany). The WSA and tablets “B” and “C” were prepared by direct compression according to the methodology described previously [27].

#### 2.1.2. Preparation of Binary Mixtures (Warfarin/Excipient)

For the study of warfarin/excipient interactions, binary mixtures of WSC or WSA with Di-Cafos^®^ 92-14, microcrystalline cellulose (MCC), starch, colloidal silica and lactose monohydrate (Tablettose^®^ 80) were prepared by mixing the individual components in a 1:3 ratio (*w*/*w*, warfarin/excipient). The ratio 1:3 was chosen as acceptable compromise when the amount of excipient remains significantly high to considerably affect the active compound and promote structural and physicochemical changes. Simultaneously, the amount of the active compound is still sufficient to observe the induced structural changes spectroscopically with high signal-to-noise ratio in acceptable experimental time.

### 2.2. Stability Studies

Tables A, B, and C and the binary mixtures were subjected to stability studies. The stability studies were carried out at a temperature of 40 °C and 75% RH (accelerated stability test) in a constant climate chamber (Binder, type KBF 115/240, Germany). The commercial tablets (sample “A”) were left in the original packaging (blister), and samples B and C were placed in 30 mL polypropylene containers, which were closed. Dissolution profiles after a storage time of 6 months were compared for the accelerated stability test. The same stability tests were also performed for neat WSC, WSA and the corresponding binary mixtures. The structural changes induced by aging during the stability studies were monitored by solid-state NMR spectroscopy and Raman spectroscopy.

### 2.3. Physicochemical Testing of Tablets

Tablet thickness, diameter, crushing strength and weight (*n* = 10 tablets) were measured automatically on a Pharmatest WHT-1 (Pharmatest, Hainburg, Germany). Mass uniformity was evaluated according to Ph. Eur. 2.9.5, and the tablets’ resistance to crushing was determined according to Ph. Eur. 2.9.8. Friability was tested according to Ph. Eur. 2.9.7 using a TAR 10 friability tester (Erweka, Langen, Germany). Disintegration was tested according to Ph. Eur. 2.9.1 using a ZT 4 tablet disintegration tester (Erweka, Langen, Germany). The determination of the contents was performed by HPLC (Agilent 1260, USA), and the sample treatment was performed according to a previous paper [28].

### 2.4. In Vitro Drug Release

#### 2.4.1. Dissolution Test of Tablets

Dissolution tests were performed using a SOTAX (AT7 Donau Lab, Uster, Switzerland) device. Either distilled water (the methodology for warfarin tablets described by USP) or phosphate buffer at pH 6.8 (the methodology recommended by the FDA for warfarin tablets) was used as a dissolution medium [29]. The volume of the dissolution medium was 900 mL, and the temperature was kept constant at 37.0 ± 0.5 °C. The test was performed using a Ph. Eur. 2.9.3 Apparatus II (paddles, 50 rpm). Six tablets were used for each test. The dissolved amount of the active substance was determined by HPLC at the following time intervals: 5, 10, 20, 30, 60, and 120 min [28].

#### 2.4.2. Biphasic Dissolution Test of Tablets

Biphasic dissolution was performed using 900 mL of 0.1 mol L^–1^ HCl and 70.0 g of 1-octanol maintained at 37.0 ± 0.5 °C using Apparatus II (paddles, 50 rpm). Six tablets were used for each test. One milliliter of dissolution medium (water and organic phase) was withdrawn after 1, 2, 3, 4, 6 and 8 h of dissolution. Samples from all dissolution tests were filtered using 0.45-μm syringe filters; the warfarin content was then analyzed by HPLC.

#### 2.4.3. Dissolution Test of Non-Ionized Warfarin (Acid Form) with Different Particle Sizes

Two grams of the acid form of warfarin (Sigma Aldrich, UK) was dissolved in 150 mL of methanol, evaporated, crushed in a mortar blender and passed through sieves with 250-μm and 80-μm mesh sizes. Five milligrams of warfarin (fractions above and below the 80-μm mesh) was manually filled into a hard, gelatinous capsule size 0 (Dr. Kulich Pharma s.r.o., Czech Republic) and subsequently replenished with lactose (Pharmatose^®^ 200M, DMV Int., the Netherlands) up to 200 mg of total weight. Six capsules were prepared from each fraction (above and below the 80 μm mesh sieve). To eliminate the disintegration time of capsule bodies, the spherical canopy of each capsule was cut off with a razor before testing. Subsequently, the dissolution test was performed according to the procedure described in Section 2.4.1.

### 2.5. Warfarin Solid State Characterization

#### 2.5.1. Solid-State NMR Spectroscopy

Solid-state NMR spectra were measured at 11.7 T using a Bruker Avance III HD 500 WB NMR spectrometer (Karlsruhe, Germany, 2013) with a 3.2 or 4 mm probehead in ZrO_2_ rotors. The ^13^C CP/MAS NMR spectra employing cross-polarization (CP) were acquired using the standard pulse scheme at a spinning frequency of 11 or 15 kHz. The optimized recycling delay was 6 s, and the cross-polarization contact time was 2 ms. The strength of the spin-locking fields had a *B*_1_(^13^C) field nutation frequency of 62.5 kHz. The number of scans was 1–30 k. Single-pulse ^23^Na and ^31^P MAS NMR spectra were acquired at a spinning frequency of 15 kHz with a pulse width of 1.0 μs (30° flip angle); excitation field intensity *B*_1_(^23^Na, ^31^P) of 83.3 kHz; recycle delays of 2 and 20 s, respectively; and the number of scans 64-1024 depending on the signal-to-noise ratio. Frictional heating [30] of the spinning samples was compensated by active cooling, and the temperature calibration was performed with Pb(NO_3_)_2_.

#### 2.5.2. Raman Scattering Measurements

Raman scattering measurements were performed using a NanoFinder S confocal micro-Raman microscope (SOL instruments, Minsk, Belarus). The Raman scattering spectra were excited at 633 nm with 10 mW power. The system was calibrated on silicon (520.8 cm^–1^). The beam was focused on the samples with a 50× microscope objective with a numerical aperture of 0.8. The exposure time was 30, 60 s and 120 s with a grating with 2.7 cm^–1^ resolution. All measurements were performed at room temperature in an ambient atmosphere.

#### 2.5.3. Particle Size

The particle/agglomerate size of the powdered warfarin samples was assessed via laser diffraction (Helos KR, Sympatec GmbH, Clausthal-Zellerfeld, Germany). Samples were analyzed in an aerodispersion, with 2-bar-pressurized air used to deagglomerate them. The sample analysis was performed 3 times with the Helos program that defined a statistical algorithm for data evaluation.

## 3. Results and Discussion

### 3.1. API Solid-State Change Characterization in Tablets

In general, there are a range of spectroscopic methods that allow monitoring of structural changes and transformations of active pharmaceutical ingredients (APIs) at the atomic resolution level [31,32]. Among them, X-ray powder diffraction (XRPD) is considered to be the gold standard because it is a robust, easy-to-implement and usually cost-effective technique. Moreover, quite recently, advanced techniques for complete crystal structure determination of powdered materials have been developed; these techniques can even be used for large and complex compounds [33,34]. However, the small content of APIs and the presence of crystalline excipients in drug formulations complicate the interpretation of XRPD patterns. As the XRPD record is quantitative, the reflections of APIs often overlap with the signals of predominating excipients [31]. In contrast, ssNMR spectroscopy provides considerably higher selectivity. In ^13^C CP/MAS NMR spectra, the spectral regions specific for an active compound or excipients can be found, thus allowing easy identification of the structural and physical state of an active compound and excipients. Moreover, even if the signals overlap in ^13^C CP/MAS NMR spectra, one can record NMR spectra of other nuclei that can provide much higher selectivity. Recently, we demonstrated the high potential of ^19^F MAS NMR spectra for monitoring the extreme polymorphisms of atorvastatin in tablet formulations [31,35]. In our particular case, such suitable nuclei were ^23^Na or ^31^P.

#### 3.1.1. Commercial Tablets

**Scheme 1:**
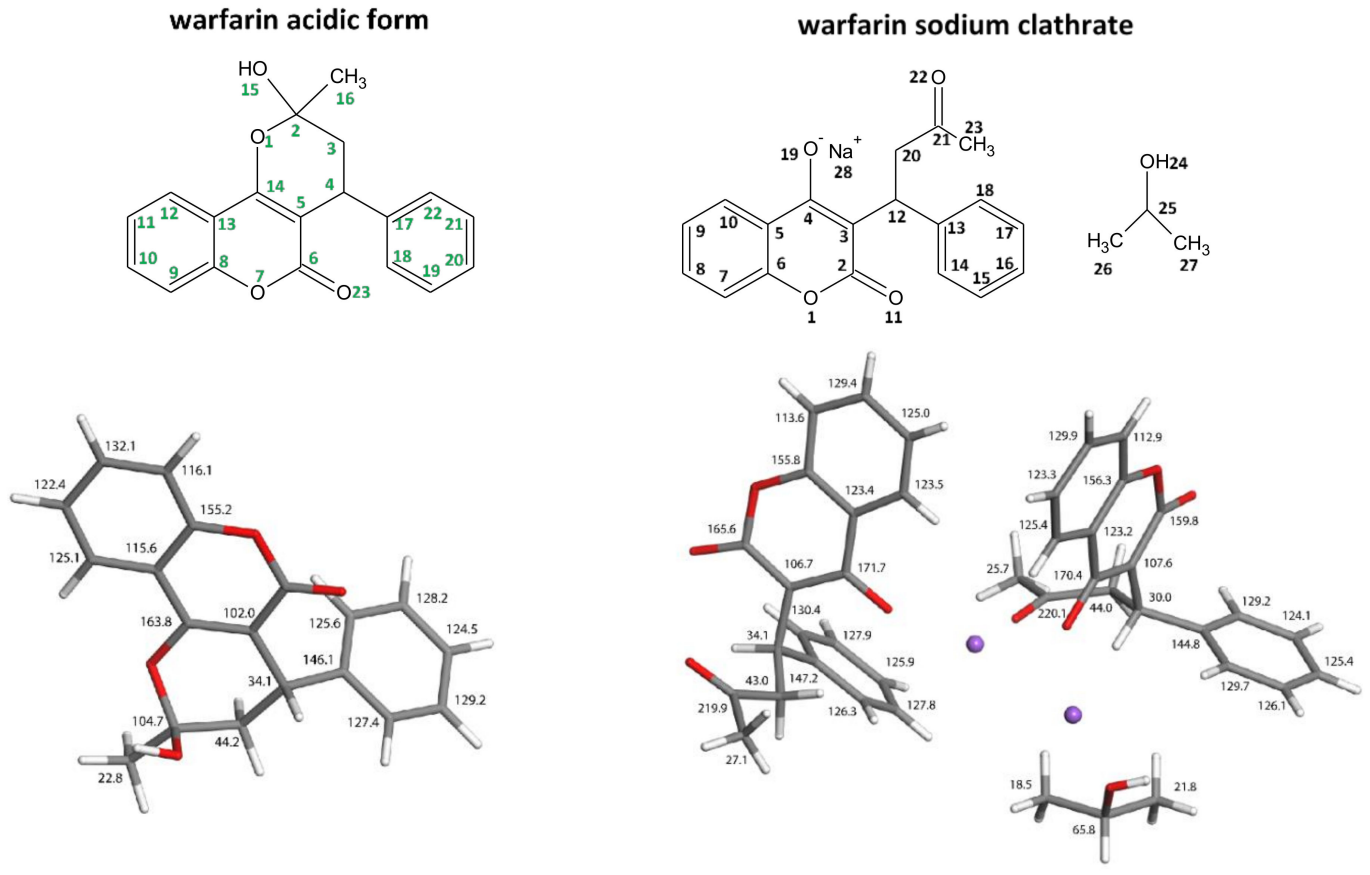
Chemical structures of the acidic (cyclic) form of warfarin (WA) and warfarin sodium clathrate (WSC or WSA) and the corresponding DFT optimized geometries, which are shown together with the ^13^C isotropic chemical shielding values calculated by the GIPAW-PBE approach as described in our previous work [38].

**Figure 1:**
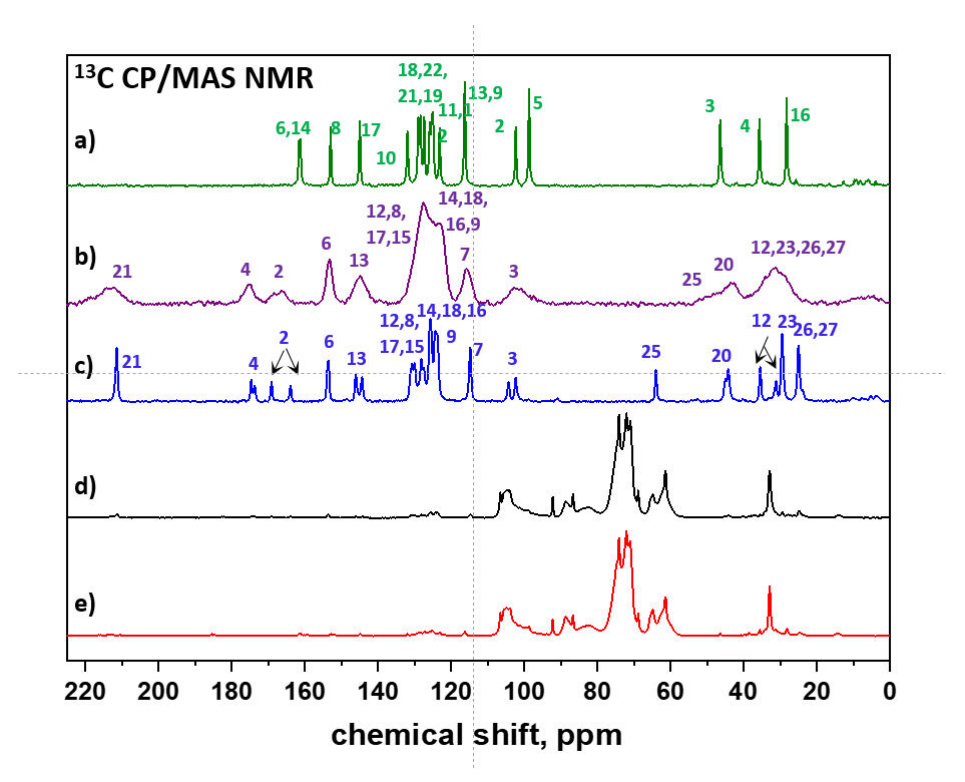
^13^C CP/MAS NMR spectra of the acid (cyclic) form of warfarin (WA) (**a**); warfarin sodium amorphous (WSA) (**b**); warfarin sodium clathrate crystalline (WSC) (**c**); commercial tablets “A” before (**d**); and after stability study (**e**).

To obtain clear benchmarks for reliable monitoring of the structural stability of commercially available products, in the first step, we recorded the ^13^C CP/MAS NMR spectra of the pure forms of warfarin (Scheme 1) in all available physical states (Figure 1a–c; WA, WSA, WSC, respectively). The signal assignment was performed on the basis of density functional theory (DFT) calculations and our previous experience (Scheme 1). As indicated by the narrow signals of pure WA and WSC (the linewidth is ca. 50–100 Hz, Figure 1a,c), the original, unmodified active substances are highly crystalline systems without structural disorder. In the case of WSC, the molar ratio of warfarin:IPA was determined to be 2:1 (Figure 1c), which is in accord with literature data for this polymorphic form [36,37]. The signals of the amorphous form are considerably broadened (Figure 1b).

**Figure 2:**
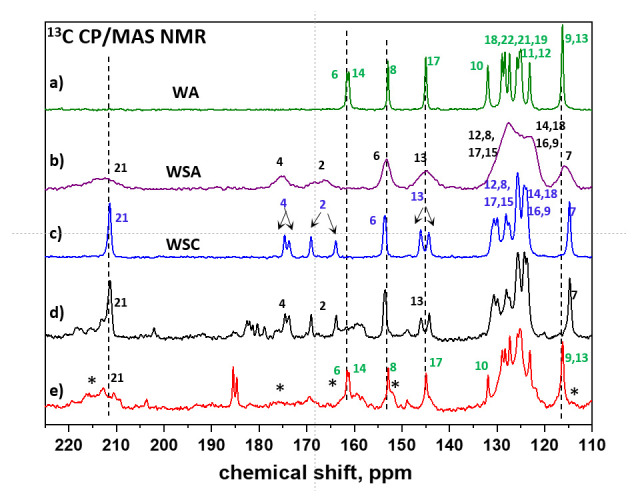
The expanded region of 225-110 ppm of ^13^C CP/MAS NMR spectra of the acid (cyclic) form of warfarin (WA) (**a**); warfarin sodium amorphous (WSA) (**b**); warfarin sodium clathrate crystalline (WSC) (**c**); commercial tablets “A” before (**d**); and after stability study (**e**). The signals marked by asterisk * indicate the presence of amorphous sodium form of warfarin (WSA).

As the amount of warfarin in commercial tablet A was only 3%, the total acquisition time of a single ^13^C CP/MAS NMR spectrum had to be at least 72 h to reach an acceptable signal-to-noise ratio and to clearly detect key NMR signals of warfarin, allowing identification of its structural and physical state (Figure 1d,e and Figure 2d,e). Subsequently, analysis of the high-frequency region from ca. 120 to 200 ppm, in which there is no risk of overlap with the signal of the excipient, then confirmed the presence of the crystalline sodium clathrate form of warfarin (WSC) in the investigated commercial tablets (Figure 2d). This form is clearly indicated by the narrow signals of the ketone unit ca. 212 ppm and Na–O–CH= and O–C=O carbons (#4 and #2) resonating ca. 175–165 ppm. In addition, the crystalline fraction is accompanied by a fraction of an amorphous or disordered phase, which is indicated by the broad signals ca. 215 ppm. Accelerated aging, however, induced substantial structural transformation of the API. The crystalline sodium clathrate form of warfarin (WSC) predominantly changed to the amorphous sodium form of warfarin (WSA), which is indicated by the disappearance of the corresponding narrow signals and their notable broadening (see the signals marked by asterisks in Figure 2e). In addition, the second fraction of warfarin was transformed to the crystalline acid form (WA), as indicated by the narrow signals ca. 162, 153 and 145 ppm, whereas the third fraction adopted the corresponding acidic amorphous form, which is indicated by the broad signals resonating nearly at the same positions (see the signals marked by arrows in Figure 2e). The overall crystallinity of warfarin estimated from the signal intensities was, however, very low at 15–10%, whereas amorphous fractions occupied more than 85%. The signal ca. 185 ppm is attributed to the sodium carbonate group O=C–ONa [39], probably located in the cellulose polymer chains.

**Figure 3:**
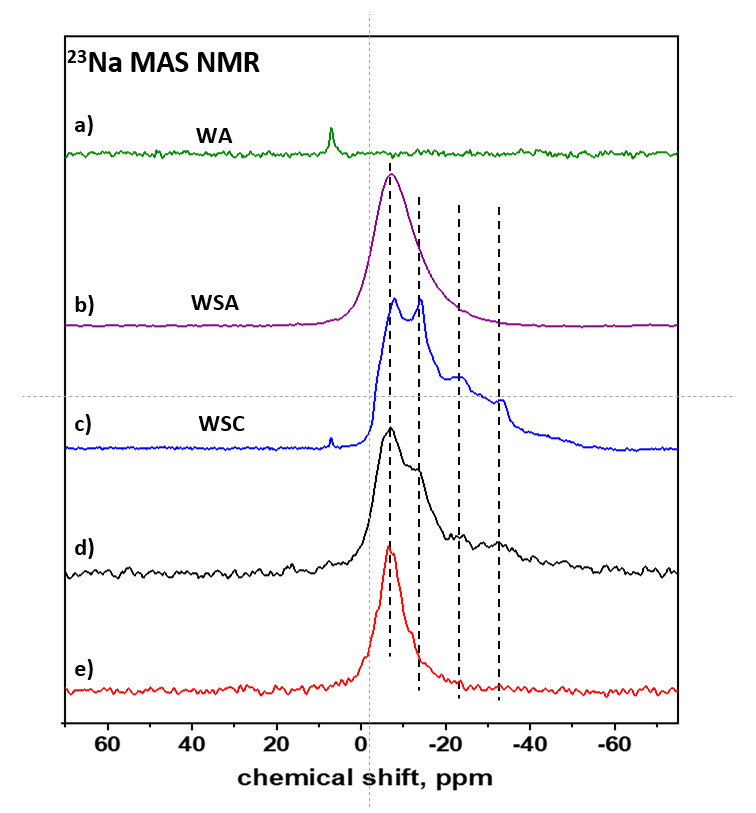
A: ^23^Na MAS NMR spectra of the acid (cyclic) form of warfarin (WA) (**a**); warfarin sodium amorphous (WSA) (**b**); warfarin sodium clathrate crystalline (WSC) (**c**); commercial tablets “A” before (**d**); and after stability study (**e**). The signal at 7.1 ppm corresponds to NaCl.

Owing to the low sensitivity of ^13^C CP/MAS NMR spectroscopy, which can be problematic, especially in the analysis of low-dose tablet formulations, we decided to test the application potential of ^23^Na MAS NMR spectroscopy, which offers stronger signals due to the 100% natural abundance of ^23^Na isotopes at considerably shorter experimental times. Clearly, while the acidic form of warfarin has no sodium ion in its molecule (Figure 3a), amorphous sodium warfarin (WSA) is characterized by a broad featureless resonance centered at −7.1 ppm with a halfwidth of 1545 Hz (Figure 3b). In contrast, the crystalline sodium warfarin clathrate (WSC) exhibits a typical quadrupolar spectral pattern with a range of sharp maxima and edges specifically at −7.7, −14.2, −23 and −33.4 ppm (Figure 3c). The spectral pattern obtained for tablet formulation A before the stability test clearly exhibits a two-component character, from which it follows that crystalline sodium warfarin clathrate (WSC) is accompanied by its amorphous fraction (Figure 3d). After accelerated aging, the recorded ^23^NaMAS NMR resonance significantly narrowed to a single signal resonating at -6.5 ppm with a linewidth of 928 Hz. The observed disappearance of the low-frequency resonances at −23.4 and −32.2 ppm confirms the complete transformation of the crystalline warfarin sodium clathrate. The narrowing of the central signal to ca. 928 Hz then indicates increased mobility of the sodium ions, which are partly incorporated in the domains of amorphous sodium warfarin. In addition, a significant fraction of sodium ions released by transformation of sodium warfarin to its acidic form migrated to the hydrated cellulose matrix. All these findings, which are in accord with the ^13^C CP/MAS NMR results, thus clearly confirm the application potential of ^23^Na MAS NMR spectroscopy for reliable analysis of tablet formulations of sodium derivatives of active pharmaceutical substances.

**Figure 4:**
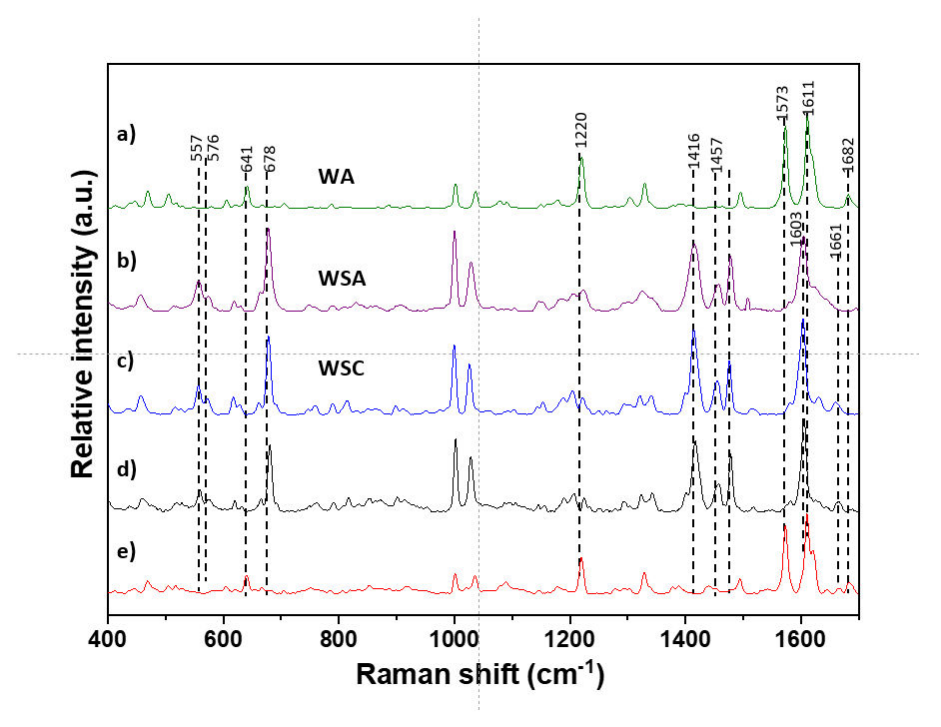
Raman spectra of the acid (cyclic) form of warfarin (WA) (**a**); warfarin sodium amorphous (WSA) (**b**); warfarin sodium clathrate crystalline (WSC) (**c**); commercial tablets A before (**d**); and after stability study (**e**).

The transformation of warfarin sodium clathrate crystalline in commercial tablet A to the acid form of warfarin during accelerated aging was also confirmed by Raman spectroscopy. Figure 4 shows the Raman spectra of native forms of warfarin (4a,b,c—acid form of warfarin; warfarin sodium amorphous; warfarin sodium clathrate crystalline, respectively), as well as the spectra of commercial tablets before (Figure 4d) and after stability study (Figure 4e). Specifically, the transformation of crystalline warfarin sodium clathrate to its acidic form is clearly reflected by the changes in the signals ranging from 1550 to 1650 cm^−1^.

Overall, all the performed experiments unambiguously revealed conversion reactions of crystalline sodium warfarin clathrate under the accelerated stability test. It is clear that some warfarin sodium in the dosage form can be transformed to the nonionized (acidic) form, probably due to the interaction with excipients. As this acidic form of warfarin is poorly soluble, its formation in tablet formulations may affect the dissolution profile and bioavailability of the active substance. These findings thus initiated a subsequent investigation of model systems, which allowed a better understanding of warfarin-excipient interactions.

#### 3.1.2. Model Warfarin Tablets

**Figure 5:**
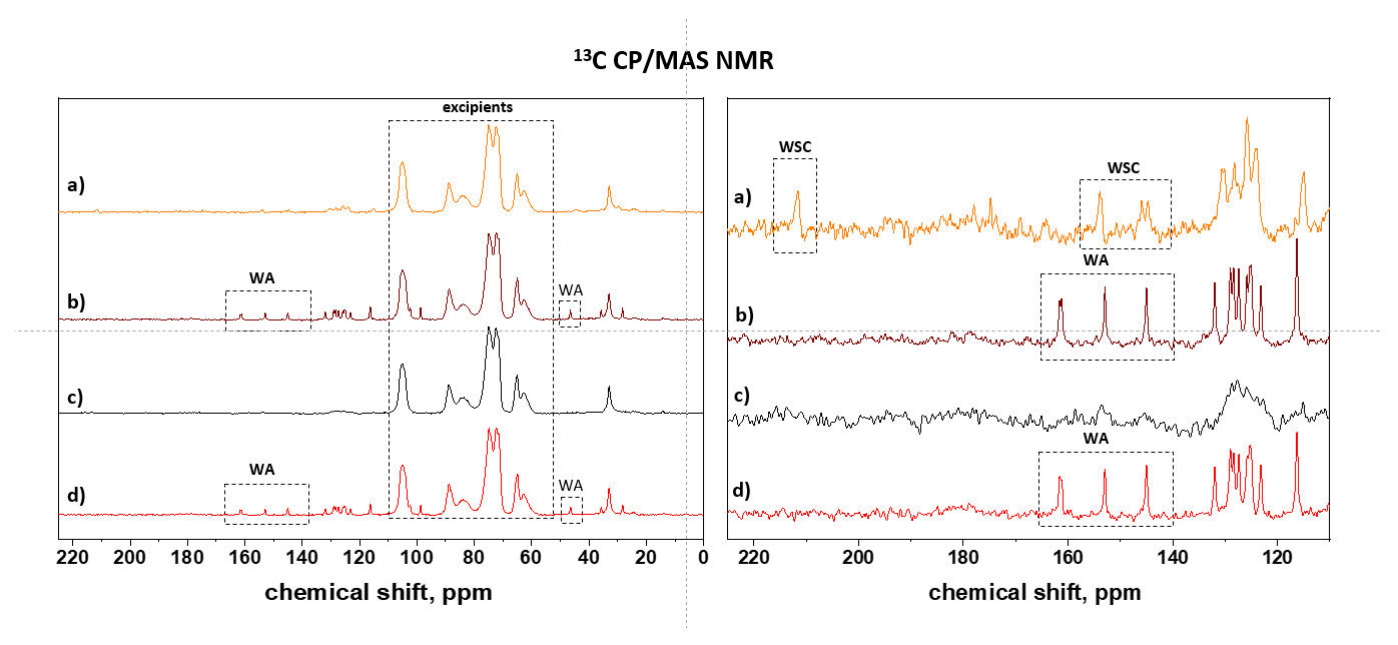
The ^13^C CP/MAS NMR spectra (left) and the expanded region of 225-110 ppm (right) multiplied by 4 of ^13^C CP/MAS NMR spectra of tablet B before (**a**); and after stability study (**b**); tablet C before (**c**); and after stability study (**d**).

**Figure 6:**
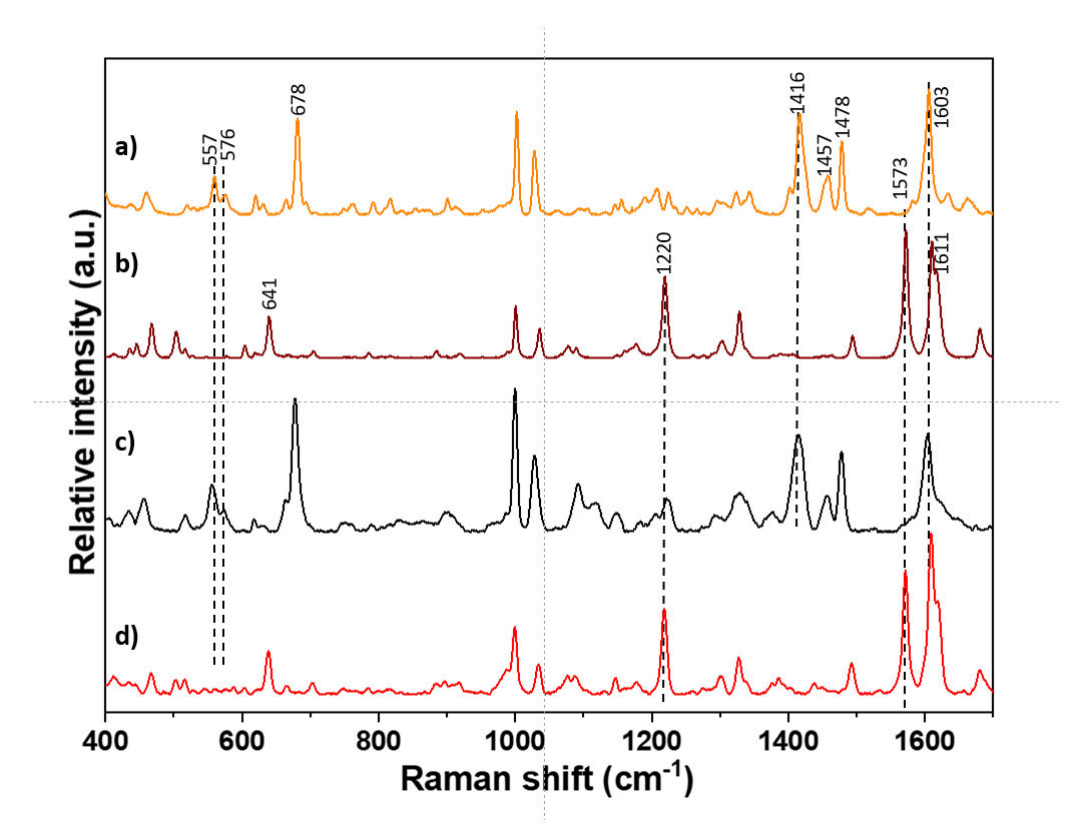
The Raman spectra of the WSC tablets (samples B) before (**a**) and after stability study (**b**); and WSA tablets (samples C) before (**c**) and after stability study (**d**).

On the basis of the above-documented solid-state transformations of warfarin in commercial tablets, model systems (tablets B and C) with precisely defined and controllable compositions were prepared. Specifically, tablets B and C were prepared with either WSC or WSA as the active compound, respectively, whereas Di-Cafos^®^ 92-14, Avicel^®^ PH 101, Ac-Di-Sol^®^ and a magnesium stearate were used as excipients [27]. The ^13^C CP/MAS NMR spectra recorded before and after the accelerated stability study (Figure 5) clearly revealed that the crystalline and amorphous forms of warfarin sodium (WSC or WSA) completely convert to crystalline acidic warfarin when interacting with the excipient. This fact, which is unambiguously reflected by the narrow signals at ca. 162, 153 and 145 ppm (Figure 5b,d), clearly indicates a high tendency of the sodium forms of warfarin to interact with some types of excipients and to undergo phase transitions. The Raman spectra of the tablets (samples B and C) before and after the accelerated stability study confirmed the described transformation of WSA and WSC to the acid form of warfarin (Figure 6).

In this way, the bioavailability of this active compound can be significantly reduced [23]. Therefore, before the detailed investigation of API-excipient interactions, we first evaluated the impact of the observed structural changes on the physicochemical properties and in vitro dissolution and whether they can potentially affect the bioequivalence.

### 3.2. Physicochemical Characterization of API and Tablets

First, the physicochemical stability of the tablets during storage under accelerated stability conditions was evaluated based on the pharmacopoeial requirements, and as demonstrated in Table 1, all the investigated tablet specimens met the requirements of Ph. Eur. (see Section 2.3). Moreover, the tested parameters did not change significantly during storage under the conditions of the accelerated stability study. Only the hardness of tablets A decreased slightly, which corresponds to the typical behavior of directly compressed tablet blends observed during stress tests [40].

The particle size distribution of API is another important parameter in dosage form development and formulation. Particularly for poorly soluble drugs, the particle size has a significant effect on the rate of dissolution. Consequently, we first determined the particle size distribution of the original powdered substances WSC and WSA used for the preparation of model tablets. The laser diffraction results summarized in Table 2 show significant differences between the powdered substances, as the WSC particles exhibit a considerably smaller average diameter of d_50_ = 12.9 μm; in contrast, the WSA particles have an average d_50_ diameter of 60.7 μm. When warfarin is in the form of a very soluble sodium salt, the different particle size distributions of WSC and WSA have no significant effect on the dissolution profile. However, after the transition to the poorly soluble WA form, the different size distributions of the parent WSC and WSA particles may lead to altered dissolution behavior.

### 3.3. Dissolution Tests

To explore the changes in bioavailability, three different dissolution tests were used to increase the robustness of the results obtained in vitro and to predict the dissolution of the API in vivo. The methodology for warfarin tablets recommended by USP (medium water), that recommended by the FDA (medium phosphate buffer at pH 6.8) and the biphasic dissolution method, which simulates an acidic gastric environment and the lipophilic character of cell membranes (0.1 M HCl/octanol) were used [41].

#### 3.3.1. Commercial Tablets

**Figure 7:**
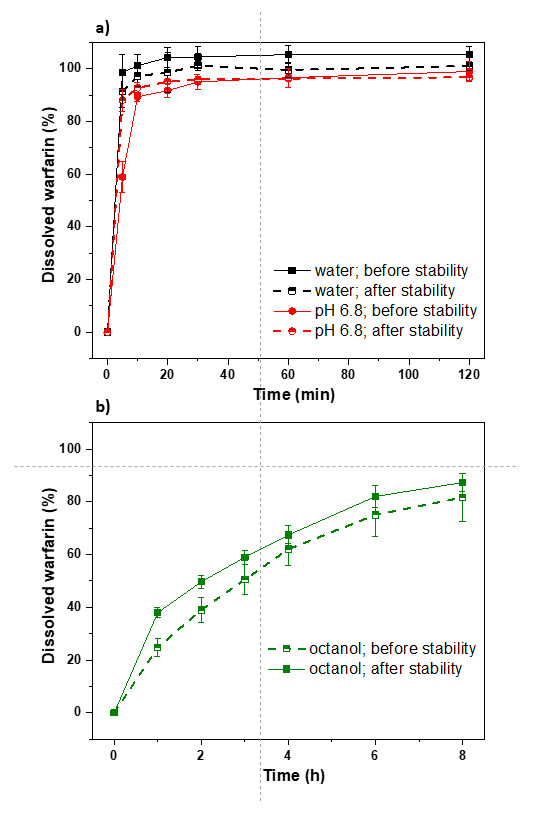
The dissolution profiles of commercial tablets “A” before and after accelerated stability study in different dissolution media: water and phosphate buffer pH 6.8 (**a**); biphasic dissolution—octanol phase (**b**).

In aqueous media (pH 6.8 and water), more than 85% of the API from the commercial tablets dissolved within 15 min (Figure 7a), so the profiles may be accepted as similar without further mathematical evaluation. In contrast, in the biphasic dissolution media (Figure 7b), the similarity of dissolution profiles was evaluated by calculating the similarity factor *f*_2_ [42,43]. In this case, the calculated value of the similarity factor *f*_2_ for the tablets “A” is 52.6, which suggests that the recorded dissolution profiles before and after the accelerated stability test are similar (*f*_2_ > 50). These findings thus clearly indicate that the observed transition of WSC to the amorphous form and partly to WA has no significant impact on in vitro dissolution. This fact can be explained by fact that only ca. 15% of the API was converted to the poorly soluble WA during the accelerated stability study.

#### 3.3.2. Model Warfarin Tablets

**Figure 8:**
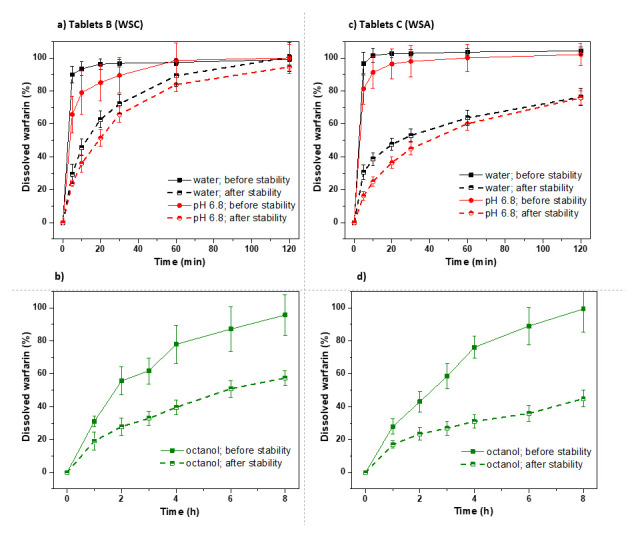
The dissolution profiles of model tablets “B” before and after accelerated stability study in different dissolution media: water and phosphate buffer pH 6.8 (**a**); biphasic dissolution—octanol phase (**b**); the dissolution profiles of model tablets “C” before and after accelerated stability study in different dissolution media: water and phosphate buffer pH 6.8 (**c**); and biphasic dissolution—octanol phase (**d**).

In contrast to the commercial tablets, the WSC and WSA in the model tablets were completely transformed into the acid form during the accelerated stability study. Consequently, this change significantly affected the dissolution properties, as clearly demonstrated in Figure 8. In any medium, the dissolution profiles indicate considerably slower dissolution of the active compounds after the stability tests. Specifically, for biphasic dissolution, the values of the similarity factor *f*_2_ calculated for tablets “B” and “C” are clearly less than 50 and reach only 26.1 and 22.4, respectively.

Moreover, the obtained data also clearly indicate that tablets “B”, which were prepared from the crystalline substance WSC, exhibit considerably faster dissolution after the stability test than tablets “C”, which were prepared from the amorphous form WSA. This counterintuitive phenomenon was observed after the stability tests in all media (water, phosphate buffer at pH 6.8 and biphasic dissolution). Because the physicochemical properties of tablets “B” and “C” did not differ significantly after the stability test (Table 1), and the API was nearly completely transformed to the identical acid form of warfarin (WA), a possible explanation for the observed differences in API dissolution lies in the differences in the particle size distribution of the original substances used to produce the tablets. As the particle sizes of the original WSC and WSA substances significantly differ (Table 2), we suppose that the post-stability tablets also contain particles of active compounds of different sizes. Specifically, it can be supposed that particles of the acidic form of warfarin (WA) in tablets “B” are considerably smaller than those in tablets “C” after the stability test. As a result, of the smaller particle size, tablets “B” exhibit considerably faster dissolution of the active compound (warfarin acidic form) after the stability test.

**Figure 9:**
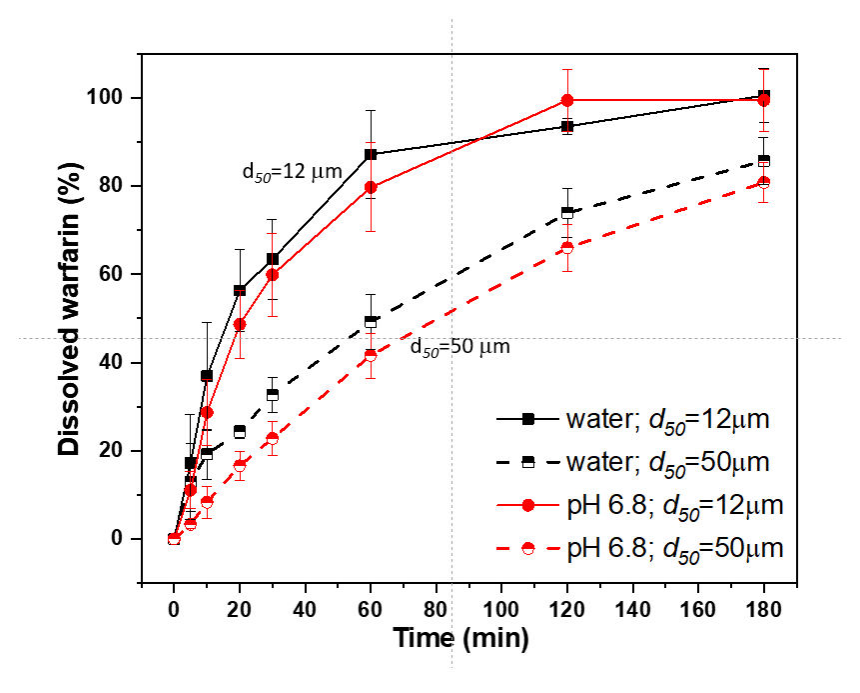
The dissolution profiles of nonionized (acid) warfarin at two different particle sizes.

The suggested effect of the particle size of the warfarin acidic form on dissolution was experimentally verified, as demonstrated in Figure 9. Capsules containing WA with a smaller particle size (d_50_ = 11.9 µm) dissolve significantly more quickly than capsules containing WA, which has a larger particle size (d_50_ = 50.0 µm). All the obtained findings thus clearly demonstrate the key role of particle size distribution in controlling the dissolution and bioavailability of poorly soluble active compounds.

In general, from a safety perspective, the transition of warfarin to the acid form may be risky if the particle size in the generic preparations differs significantly. Bioequivalent tablets may exhibit differences in the dissolution rate and API adsorption in vivo when the warfarin sodium form is converted to the acid form as it expires. This may have been the cause of bleeding in generic substitutions in the past [44]. Therefore, the appropriate choice of excipients is essential for increasing the safety of the treatment and minimizing any transition to a poorly soluble form.

### 3.4. Warfarin-Excipient Interactions in Binary Mixtures

To better understand warfarin-excipient interactions, to find a key initiator responsible for the API phase transitions, and hence to identify suitable and safe excipients for sodium warfarin clathrate, we prepared a representative set of binary systems, including microcrystalline cellulose (MCC), starch, lactose, colloidal silica and Di-Cafos^®^. All the prepared binary systems with an API/excipient composition of 1:3 (*w/w*) were subjected to stability tests, and the corresponding ^13^C CP/MAS NMR spectra were collected.

**Figure 10:**
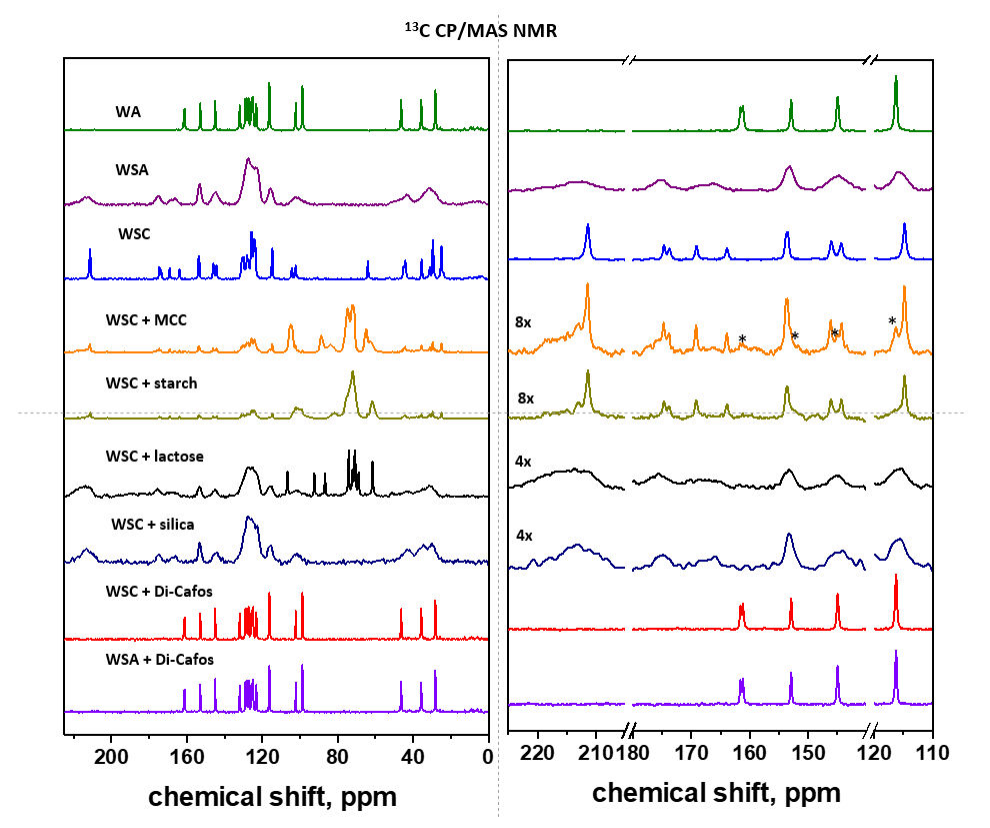
^13^C CP/MAS NMR spectra of the acid (cyclic) form of warfarin—WA; warfarin sodium amorphous—WSA; warfarin sodium clathrate crystalline—WSC and binary systems WSC—microcrystalline cellulose (MCC); WSC-starch; WSC-lactose; WSC-colloidal silica; WSC-Di-Cafos and WSA–Di-Cafos.

As demonstrated in the ^13^C CP/MAS NMR spectra recorded at the end of the testing procedure (Figure 10), the prepared binary systems exhibit three distinctly different extents of phase transformation. Minimal, nearly negligible changes were found for the WSC-starch system, for which the API remained in the original form (crystalline sodium warfarin clathrate), while nearly negligible formation of the amorphous sodium form was observed. The rate of amorphization was estimated to be less than 5%. Similar changes were also observed for the WSC-MCC system, which contained microcrystalline cellulose. In this case, however, the process of weak amorphization was accompanied by the formation of a small amount of crystalline warfarin in the acidic form. This fact is clearly demonstrated by the narrow signals at 161.7, 153, 145, and 116.3 ppm (Figure 10, signals are marked as asterisks). The WSC–lactose and WSC–colloidal silica systems exhibited the extensive, nearly complete transformation of crystalline sodium warfarin clathrate to its disordered amorphous form. This amorphization was clearly indicated by the broadening of the signal ca. 210 ppm. The high intensity and resonance frequency of this signal, reflecting the ketone carbonyl group, however, indicated the reasonable chemical stability of warfarin sodium in the lactose and colloidal silica excipients. In contrast, when Di-Cafos was used as an excipient, the crystalline warfarin sodium clathrate was completely converted to the crystalline acidic form. This process was clearly indicated in ^13^C CP/MAS NMR spectra in which the resonance frequencies ca. 210 ppm, reflecting that the ketone carbonyl group disappeared, while four narrow signals at 161.6, 161.1, 152.9, and 145 ppm indicated that the acidic form appeared (Figure 10).

**Figure 11:**
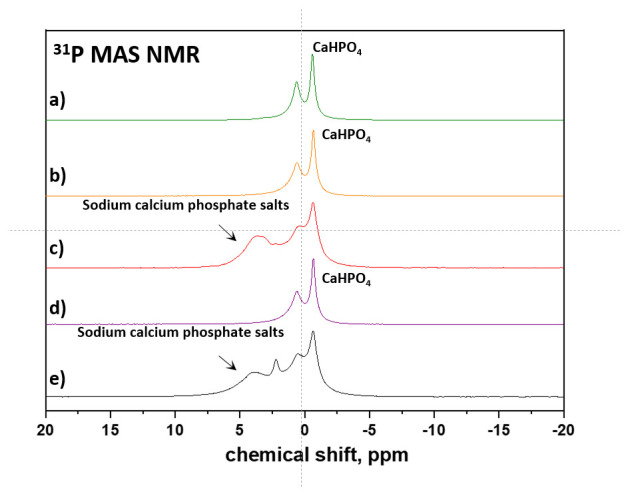
^31^P MAS NMR spectra of CaHPO_4_ (**a**); binary mixture warfarin sodium clathrate crystalline (WSC) and Di-Cafos before (**b**) and after stability tests (**c**); binary mixture warfarin sodium clathrate amorphous (WSA) and Di-Cafos before (**d**) and after stability tests (**e**).

When analyzing the considerably different extents of the API transformation and API–excipient interactions, one must consider the chemical origin and activity of the excipients used. Specifically, Di-Cafos, a CaHPO_4_ phosphate-based system, can easily participate in ion-exchange reactions [45]. Typically, the ion-exchange interaction of CaHPO_4_ with warfarin sodium can create mixed ionic species such as NaCaPO_4_ and the acidic form of warfarin. The active role of phosphate compounds in the transformation of warfarin sodium is clearly demonstrated in the ^31^P MAS NMR spectra (Figure 11), in which the formation of mixed sodium–calcium phosphate compounds is reflected by a broad signal ca. 3.6 ppm [46]. Similar ion-exchange activity can also be expected in cellulose macromolecules, for which the water penetration and moisture absorbability of the tablets increased with decreasing crystallinity of MCC through a higher level of grinding [47]. However, due to the high crystallinity of the applied microcrystalline cellulose, this ion-exchange process is generally negligible. This result is in accord with our findings for the model systems as well as for the commercial tablets.

The extensive observed amorphization of the warfarin sodium can be explained by the removal of the solvent molecules (IPA) from the crystal lattice. In particular, colloidal silica particles with their high specific surface areas can easily adsorb solvent molecules and thus induce disorder in the crystal structure. Consequently, it is thus clear that selection of an appropriate excipient for warfarin sodium requires extensive testing. In our opinion, suitable excipients should be stable, highly crystalline hydrophobic systems without reactive groups that undergo ion exchange.

## 4. Conclusions

As previously described in the literature, satisfactory content uniformity is essential for safe warfarin treatment. The crystalline state of warfarin sodium in the dosage form has no significant effect because under the conditions of the acidic environment of the stomach, the transition to the identical acidic form of warfarin occurs. A critical factor in the safety of warfarin treatment may be the observed transition of warfarin sodium to its acid form in tablets during storage because generic warfarin sodium tablets from different manufacturers may have different particle sizes. Proven interactions of APIs with excipients significantly affect the stability and dissolution rate of warfarin from tablets during storage and thus may significantly affect the bioavailability in vivo. Although definitive conclusions require extensive in vivo experiments in animal models our findings are firm and sufficiently supported by in vitro experiments and spectroscopic data. 

## Figures and Tables

**Table 1:** Physicochemical evaluation of tablets.

	Tablets A	Tablets B	Tablets C
	**Before stability study**		
API content (%)	100.6 ± 2.0	101.4 ± 1.6	98.6 ± 1.6
Mass uniformity (mg)	181.0 ± 3.8	271.5 ± 1.9	269.1 ± 1.4
Friability (%)	0.12	0.60	0.43
Disintegration (min)	less than 7	less than 1	less than 1
Hardness (N)	55.1 ± 10.9	46.0 ± 6.8	40.3 ± 8.1
Thickness (mm)	3.35 ± 0.03	2.29 ± 0.07	2.26 ± 0.01
Diameter (mm)	8.04 ± 0.01	9.97 ± 0.03	10.01 ± 0.01
	**After stability study**		
API content (%)	103.6 ± 2.8	97.8 ± 2.4	98.6 ± 3.4
Mass uniformity (mg)	180.3 ± 2.2	267.4 ± 5.1	270.0 ± 1.9
Friability (%)	0.26	0.64	0.77
Disintegration (min)	less than 5	less than 2	less than 2
Hardness (N)	36.6 ± 3.8	47.6 ± 1.4	36.8 ± 3.3
Thickness (mm)	3.46 ± 0.03	2.40 ± 0.03	2.29 ± 0.01
Diameter (mm)	8.164 ± 0.02	10.08 ± 0.01	10.10 ± 0.01

**Table 2:** Particle size distribution of WSC and WSA used for tablet production.

API Form	Particle Size [µm] ^1^		
	d_10_	d_50_	d_90_
WSC	2.3	12.9	230.3
WSA	2.1	60.7	279.0

^1^ dx = x % of measured particles are smaller than this size [µm]; WSC—warfarin sodium clathrate crystalline; WSA—warfarin sodium amorphous.

## Data Availability

A full list of references is compiled and attached to this manuscript.
